# Integrating intratumoral and peritumoral radiomics with deep transfer learning from multiparametric MRI for preoperative prediction of HER2 status in breast cancer: a multicenter study

**DOI:** 10.3389/fonc.2026.1790816

**Published:** 2026-08-28

**Authors:** Saisai Zhang, Jing Rong, Tiantian Liu, Xiujuan Yin, Shuqin Xue, Likang Yin, Lei Liu, Yang Ji, Xijun Gong, Xiao Wang

**Affiliations:** 1 Department of Radiology, The First Affiliated Hospital of Anhui Medical University, Hefei, Anhui, China; 2 Department of Radiology, Taihe People’s Hospital/Taihe Hospital Affiliated to Wannan Medical University, Fuyang, Anhui, China; 3 Department of Imaging, The First Affiliated Hospital of Xi’an Medical University, Xi’an, Shaanxi, China; 4 Department of Radiology, The Second Affiliated Hospital of Anhui Medical University, Hefei, Anhui, China

**Keywords:** breast cancer, deep transfer learning, HER2, multiparametric MRI, radiomics

## Abstract

**Purpose:**

To develop and validate a combined model integrating intratumoral and peritumoral radiomics features, deep transfer learning features from multiparametric MRI (DCE-MRI, T2WI, and DWI), and clinical indicators, and to evaluate its diagnostic performance and clinical utility for preoperative prediction of HER2 expression status in breast cancer.

**Methods:**

We collected data from 411 breast cancer patients from three centers retrospectively (training set: 212; internal validation set: 91; external test sets: 50 and 58). Multiparametric MRI (DCE/T2WI/DWI) was acquired. This study extracted manually constructed radiomics features and deep transfer-learning features based on ResNet50 from the tumor interior and peritumoral regions using multiparametric MRI. Through multiple feature-selection steps, such as intraclass correlation coefficient calculation, Spearman correlation testing, and LASSO logistic regression, a fusion feature set of deep learning and radiomics (DLR) was constructed. Finally, the DLR feature set was combined with independent clinical predictors to establish a combined prediction model. The model performance was evaluated using receiver operating characteristic curves, calibration curves, and decision curve analysis, and visual interpretability analysis was conducted using Grad-CAM and SHAP methods.

**Results:**

The combined model had the best accuracy and prediction ability, with AUC values of 0.965 (95% CI: 0.939–0.990) and 0.904 (95% CI: 0.843–0.966) for the training and internal validation cohorts, respectively. In external test sets 1 and 2, it had AUCs of 0.844 (95% CI: 0.724–0.964) and 0.846 (95% CI: 0.743–0.949), respectively.

**Conclusions:**

By integrating intratumoral/peritumoral features from multiparametric MRI, radiomics, and deep transfer learning, and combining these with clinical indicators, the developed model enables precise prediction of HER2 status in breast cancer. This provides a reliable assessment tool for precision diagnosis and treatment decisions.

## 1 Introduction

Breast cancer is the most frequent malignant tumor among women globally (1), and it is one of the main causes of cancer-related death in women. Human epidermal growth factor receptor 2 (HER2) is an important prognostic and therapeutic target for breast cancer. The proportion of patients with HER2-positive breast cancer is approximately 15%–20% of all cases, and these tumors are characterized by high malignancy and a significant recurrence risk. Anti-HER2 targeted therapies, such as trastuzumab and pertuzumab, can increase the 5-year survival rate of patients by 37% (2). Accurate assessment of HER2 status is crucial for the implementation of targeted therapy. However, at present, clinical techniques, such as immunohistochemistry (IHC) and fluorescence *in situ* hybridization (FISH), that are widely applied to determine whether a tumor overexpresses HER2 are invasive and time-consuming. Moreover, due to breast tumor heterogeneity, there is a possibility of sampling bias (3). Therefore, in clinical practice, there is a genuine need for a method that can accurately assess the overall HER2 expression level of tumors without the need for invasive tissue sampling.

Multiparametric magnetic resonance imaging (MRI) has the benefits of good soft tissue resolution, non-invasiveness, and the ability to provide multiple parameters in diagnosing breast cancer. It is also widely used to predict the diagnosis and prognosis of breast cancer (4–6). Radiomics enables the extraction of high-dimensional data that are invisible to the human eye from digital medical images in a non-invasive way to identify features related to tumor heterogeneity within regions of interest. This allows an objective assessment of the characteristics of tumors, providing a new perspective for predicting the molecular phenotype of the tumor (7, 8). However, current studies have several limitations: ① Modal monocentricity: some studies use only DCE-MRI, overlooking T2WI, which that reflects tumor edema, and DWI, which assesses cell density, resulting in substantial variation in results (9, 10); ② Regional feature limitations: traditional radiomics focus only on intratumoral features, while biological information, such as angiogenesis and inflammatory response in the peritumoral region (tumor microenvironment), correlates highly with HER2 expression (11); ③ Insufficient model generalization: previous studies on HER2 expression in breast cancer were mainly small-sample, single-center investigations lacking standard image preprocessing protocols, leading to a substantial AUC drop during external validation (12, 13).

Deep transfer learning (DTL) provides a new method for overcoming the bottlenecks of radiomics through the development of artificial intelligence technology. Recently, CNNs within deep transfer learning have been very successful in computer vision, and they have also been applied to other fields, including tumor molecular subtypes and prognostic evaluation (14, 15). Unlike radiomics, which depends on features manually defined by experts, DTL uses CNNs to automatically learn and extract features from raw image data. This approach reduces reliance on manual features and increases the accuracy of prediction models (16, 17). A study of lump-type breast cancer showed that deep learning methods had a diagnostic accuracy of 91%, an 8.3% increase over conventional radiomics (18). At the same time, a multimodal fusion strategy has shown significant advantages: DCE-MRI (hemodynamics) + T2WI (anatomical structure) + DWI (water diffusion) can comprehensively capture the tumor’s “metabolic-structural-functional” characteristics (19).

Therefore, this study aims to integrate multiparametric MRI radiomics and deep transfer learning strategies, simultaneously extract imaging features from intratumoral and peritumoral regions, and combine clinical indicators to construct a multi-source data fusion model, with the primary endpoint of evaluating the model’s efficacy in preoperative non-invasive tissue sampling prediction of HER2 status in breast cancer.

## 2 Materials and methods

### 2.1 Study population

**Figure 1:**
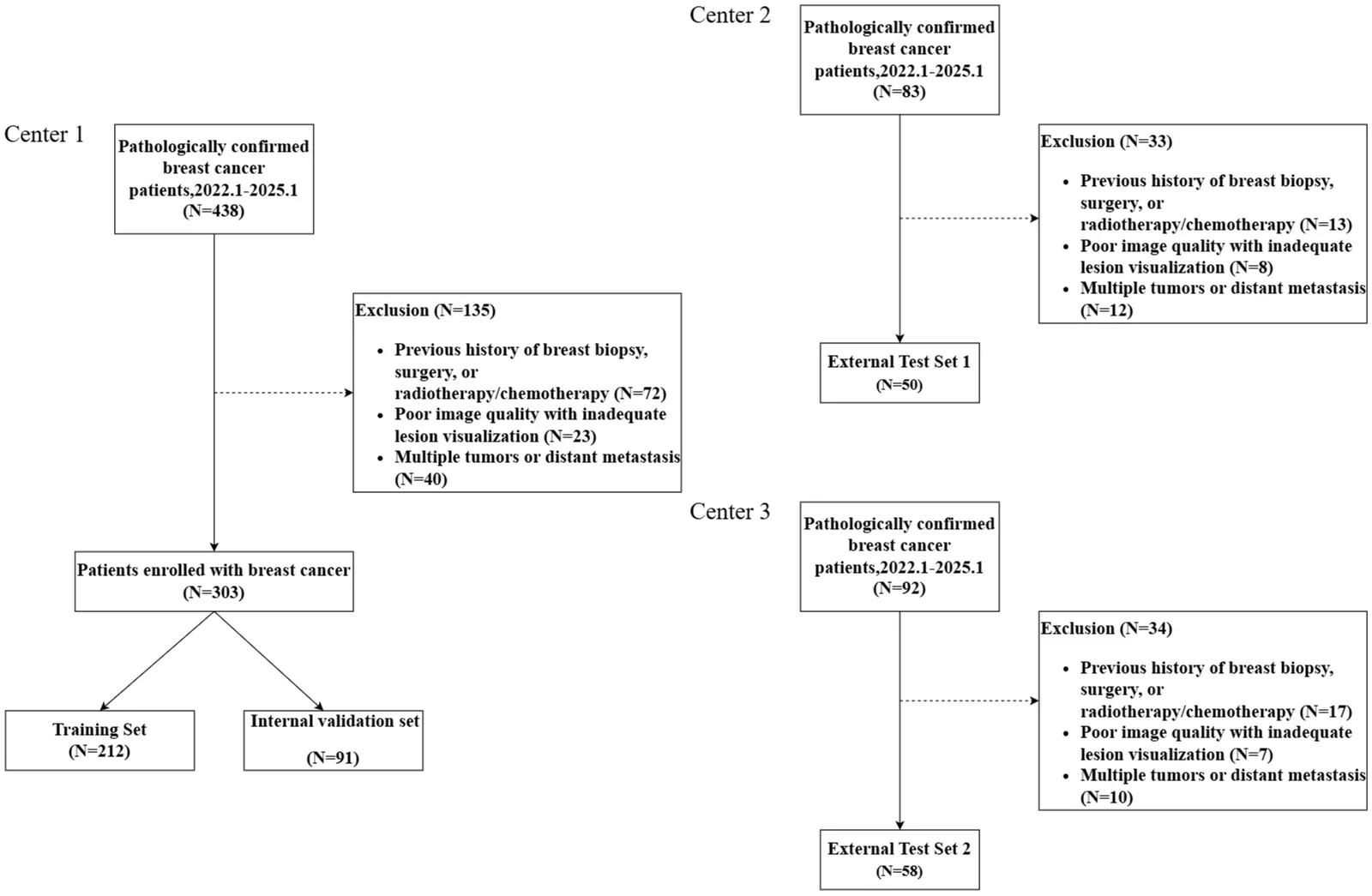
Flowchart of the patient recruitment process.

This retrospective multicenter study was granted approval by the Ethics Committee of the First Affiliated Hospital of Anhui Medical University (Ethics Approval No.: PJ-2025-05-95). In line with the Declaration of Helsinki, it was exempted from the requirement to obtain informed consent from patients. A total of 411 breast cancer patients with surgically confirmed pathology were sequentially recruited from three tertiary hospitals during the period from January 2022 to January 2025. Among them, 242 cases were HER2-positive (IHC 3+ or IHC 2+ with positive FISH result), with 122 cases in the training set, 53 cases in the internal validation set, 31 cases in the external test set 1, and 36 cases in the external test set 2; a total of 169 cases were HER2-negative (IHC 0/1+ or IHC 2+ with negative FISH result), with 90 cases in the training set, 38 cases in the internal validation set, 19 cases in the external test set 1, and 22 cases in the external test set 2. The inclusion criteria were as follows: (1) completion of multiparametric MRI within one week prior to surgery; (2) confirmation of postoperative HER2 status through the combined application of IHC and FISH testing; (3) a maximum lesion diameter of at least 5 mm; (4) no prior history of other malignancies. The exclusion criteria included: (1) previous breast biopsy, surgery, or radiotherapy/chemotherapy; (2) suboptimal image quality with insufficient lesion visualization, precluding complete lesion delineation; (3) the presence of multiple tumors or distant metastases. The inclusion and exclusion criteria, along with the screening process, are depicted in **Figure 1**.

### 2.2 MRI equipment and scanning parameters

All patients underwent conventional MRI and DCE-MRI performed on a GE Signa HD xt 3.0T MR machine with an 8-channel breast phased-array coil. All patients were lying down with both breasts over the coil. The scan for each breast was axial and sagittal. LAVA T1WI scan parameters were as follows: TR = 5.68 ms, TE = 2.20 ms, TI = 16 ms, slice thickness = 2.0 mm, slice spacing = 0 mm, FOV = 340mm × 340 mm, and matrix = 348 × 348. Contrast scanning was performed using a dual-lumen high-pressure syringe to inject a contrast agent made of diethylenetriamine pentaacetic acid (Bayer Pharmaceuticals, Germany) at a rate of 2.5mL/s, with a dose of 0.1 mmol/kg. The total time for all scans was 480 s, divided into eight phases, each lasting 60 s.

### 2.3 Image preprocessing and region of interest segmentation

**Figure 2:**
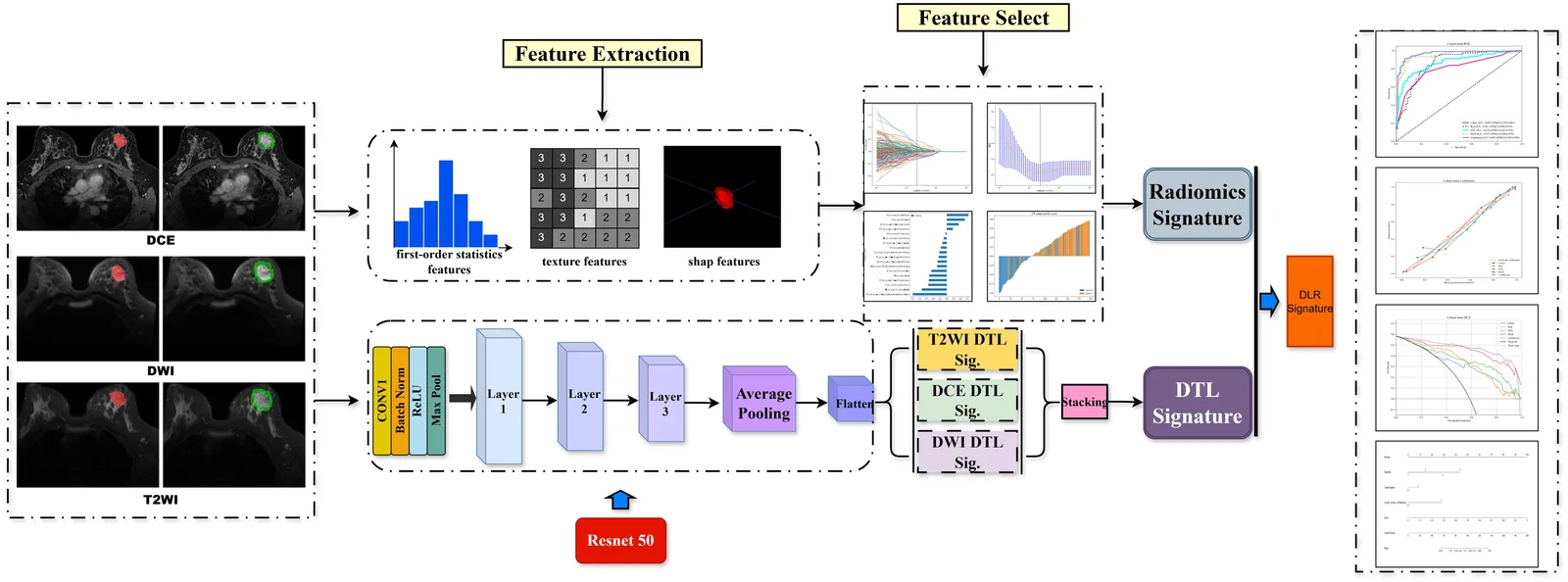
Flowchart of radiomics and deep transfer learning model construction.

MR T1-DCE phase 3 images, T2WI fat-suppression sequence images, and DWI images were imported into ITK-SNAP 3.8.0 software in DICOM format for preprocessing. The specific workflow was as follows: rigid registration–T2WI and DWI were aligned to the T1-DCE image using linear image registration tools; bias correction–N4 bias field correction was applied to all images via the “SimpleITK” software package. All patients underwent anonymization. Two radiologists with 5 and 10 years of breast MRI diagnostic experience manually segmented the tumor parenchyma layer by layer (avoiding areas of tumor liquefaction or necrosis) and used the software to automatically delineate the peritumoral region (5-mm margin) to construct the total volume of interest (VOI). Interobserver agreement was measured by the Dice similarity coefficient (DSC). The overall flowchart for this study is shown in **Figure 2**.

### 2.4 Feature extraction

Radiomics feature extraction: after manually drawing the tumor region of interest (ROI) using ITK-SNAP, we used the open-source Pyradiomics software package to extract radiomics features. The feature distribution for each sequence included shape/size features (N = 14), first-order features (N = 234), and second-order or higher-order texture features (N = 949). Feature extraction followed the standard definitions of the Image Biomarker Standardization Initiative. The consistency of radiomics feature extraction between the two doctors was evaluated by calculating the intraclass correlation coefficient (ICC). Features with a consistency coefficient lower than 0.80 were excluded.

Deep transfer learning feature extraction: the study first chose the maximum tumor cross-sectional area from the images of 411 breast cancer patients who were included in the study (which included both intratumoral and peritumoral regions). Clear definitions have established: the intratumoral volume of interest (intratumoral VOI) represented the enhanced lesion parenchymal area. The 5-mm peritumoral volume of interest (5-mm peritumoral VOI) represented the 5-mm-wide peritumoral region extending outward from the tumor boundary, which contains tumor microenvironment information. Gray values were normalized to [-1,1] with min-max transformation. Each subregion image was cropped and resized to 224 × 224 using nearest-neighbor interpolation to produce images that could be used as inputs to the model. Pretrained CNN models ResNet 50, ResNet 101, and DenseNet 121 were selected for comparative analysis in order to choose the most suitable algorithm for the specific research needs. The **Supplementary Notes** detail the complete workflow of deep transfer learning. Finally, all deep features extracted from each sequence were combined to fuse them into one single composite feature vector, $features=features_{intra}⊕features_{peri}$, which forms a DTL signature.

### 2.5 Statistical analysis

Statistical analysis was performed using SPSS 26.0. We used the Shapiro-Wilk test to assess whether the clinical characteristics followed a normal distribution. According to the distribution characteristics, continuous variables were tested with the t-test or Mann-Whitney U test, and categorical variables were tested with the chi-square (χ²) test. The AUC of the ROC curve was calculated to evaluate the model’s ability to distinguish between groups. Model fit was evaluated using calibration curves, and decision curve analysis (DCA) was used to assess clinical utility. A p < 0.05 indicated a statistically significant difference.

## 3 Results

### 3.1 Clinical baseline characteristics

A total of 411 patients were finally included in this study. Baseline characteristics for all cohorts are shown in **Table 1**. A multivariate logistic regression analysis showed that tumor density, tumor microcalcifications, and axillary lymph node metastasis were independent predictive factors of HER2 expression in breast cancer patients (**Table 2**).

**Table 1:** Baseline analysis of clinical imaging characteristics across cohorts.

Features	ALL	Train	Val	Test1	Test2	p_value
Age	50.92 ± 8.99	50.60 ± 8.26	51.36 ± 9.67	51.32 ± 11.18	51.05 ± 8.53	0.898
Diameter	25.97 ± 8.44	24.98 ± 8.52	24.29 ± 8.65	28.56 ± 6.91	30.03 ± 7.33	<0.001
ER positive rate	256 (62.3)	133 (62.7)	55 (60.4)	32 (64.0)	36 (62.1)	0.932
PR positive rate	223 (54.3)	116 (54.7)	48 (52.7)	28 (56.0)	31 (53.4)	0.968
Location						0.918
Left	207 (50.36)	109 (51.42)	43 (47.25)	26 (52.00)	29 (50.00)	
Right	204 (49.64)	103 (48.58)	48 (52.75)	24 (48.00)	29 (50.00)	
Density						<0.001
a	23 (5.60)	2 (0.94)	6 (6.59)	7 (14.00)	8 (13.79)	
b	85 (20.68)	40 (18.87)	22 (24.18)	8 (16.00)	15 (25.86)	
c	246 (59.85)	138 (65.09)	47 (51.65)	30 (60.00)	31 (53.45)	
d	57 (13.87)	32 (15.09)	16 (17.58)	5 (10.00)	4 (6.90)	
Calcification						0.618
Absence	175 (42.58)	90 (42.45)	43 (47.25)	21 (42.00)	21 (36.21)	
Presence	236 (57.42)	122 (57.55)	48 (52.75)	29 (58.00)	37 (63.79)	
BI_RADS						<0.001
4a	42 (10.22)	16 (7.55)	8 (8.79)	6 (12.00)	12 (20.69)	
4b	138 (33.58)	79 (37.26)	37 (40.66)	13 (26.00)	9 (15.52)	
4c	145 (35.28)	84 (39.62)	34 (37.36)	14 (28.00)	13 (22.41)	
5	86 (20.92)	33 (15.57)	12 (13.19)	17 (34.00)	24 (41.38)	
TIC						0.958
2	245 (59.61)	128 (60.38)	55 (60.44)	29 (58.00)	33 (56.90)	
3	166 (40.39)	84 (39.62)	36 (39.56)	21 (42.00)	25 (43.10)	
Pathological_type						0.205
IDC	366 (89.05)	190 (89.62)	80 (87.91)	45 (90.00)	51 (87.93)	
ILC	28 (6.81)	14 (6.60)	4 (4.40)	3 (6.00)	7 (12.07)	
DCIS	17 (4.14)	8 (3.77)	7 (7.69)	2 (4.00)	0 (0.00)	
lymph_node_metastasis						0.083
Absence	183 (44.53)	105 (49.53)	39 (42.86)	21 (42.00)	18 (31.03)	
Presence	228 (55.47)	107 (50.47)	52 (57.14)	29 (58.00)	40 (68.97)	
Menopausal_state						0.129
Absence	153 (37.23)	72 (33.96)	34 (37.36)	26 (52.00)	21 (36.21)	
Presence	258 (62.77)	140 (66.04)	57 (62.64)	24 (48.00)	37 (63.79)	

Density-a: The breasts are almost entirely fatty; Density-b: There are scattered areas of fibroglandular density; Density-c: The breasts are heterogeneously dense, which may obscure small masses; Density-d: The breasts are extremely dense,which lowers the sensitivity of mammography; BI_RADS: Breast Imaging Reporting and Data System; TIC: Time-Intensity Curve; IDC: Invasive Ductal Carcinoma; ILC: Invasive Lobular Carcinoma; DCIS: Ductal Carcinoma In Situ.

**Table 2:** Univariate and multivariate analyses of clinical imaging characteristics across cohorts.

Features	Univariate analysis				Multivariate analysis			
	*OR_value*	*OR lower 95% CI*	*OR upper 95% CI*	*p_value*	*OR_value*	*OR lower 95% CI*	*OR upper 95% CI*	*p_value*
Age	1.007	1.003	1.012	0.008	0.980	0.956	1.006	0.203
Diameter	1.015	1.006	1.024	0.005	0.981	0.952	1.010	0.284
Pathological_type	1.091	0.671	1.775	0.768				
BI_RADS	1.101	1.048	1.157	0.001	1.087	0.780	1.514	0.680
Density	1.163	1.077	1.257	0.001	1.616	1.105	2.361	0.038
TIC	1.170	1.064	1.285	0.006	0.645	0.373	1.115	0.188
Menopausal_state	1.258	0.951	1.664	0.177				
Location	1.452	1.044	2.020	0.063				
Calcification	2.588	1.857	3.607	<0.001	2.569	1.445	4.568	0.007
lymph_node_metastasis	3.458	2.363	5.063	<0.001	3.979	2.326	6.807	<0.001

OR, odds ratio; CI, confidence interval.

### 3.2 Feature selection and model construction

**Figure 3:**
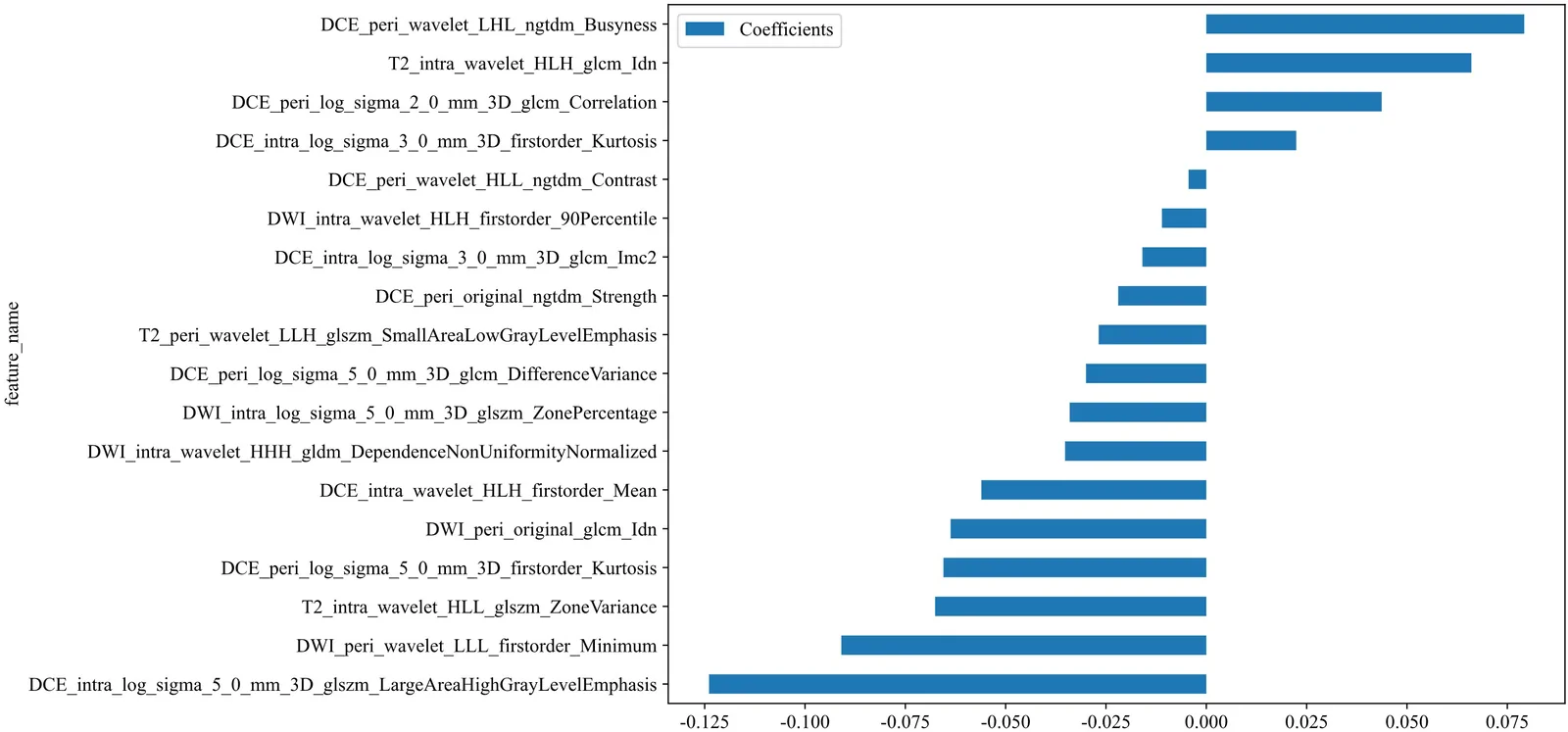
Histogram of the radiomics signature based on the selected features.

A total of 1,197 radiomics features were extracted from six VOIs over three sequences per patient (intratumoral and peritumoral regions), resulting in 7,182 features. During prefusion screening, all radiomics features underwent feature selection, and only features with non-zero coefficients were used to construct a model using the logistic regression (LR) machine-learning algorithm. The numbers and proportions of the manually selected features per group, all the corresponding P-values of all features, the 10-fold cross-validation coefficients, and the 10-fold cross-validation MSEs (mean squared errors) of all the features are presented in **Supplementary Figures 1**, **2**. The Rad_Sig histogram based on selected features is shown in **Figure 3**. Deep learning features: 2,048 features were extracted from each sequence, yielding a total of 14,336 features. Among the three models, the ResNet50 model had the best predictive performance according to **Supplementary Table 1**. By removing redundant features through Spearman rank correlation and Lasso regression, we chose the top 46 most predictive features. The logistic regression algorithm was then used to fuse filtered radiomics features and DTL features to form the deep transfer learning and radiomics (DLR) signature. Finally, there were 12 DLR features, including 5 radiomics features and 7 deep transfer learning features.

### 3.3 Model performance comparison

**Figure 4:**
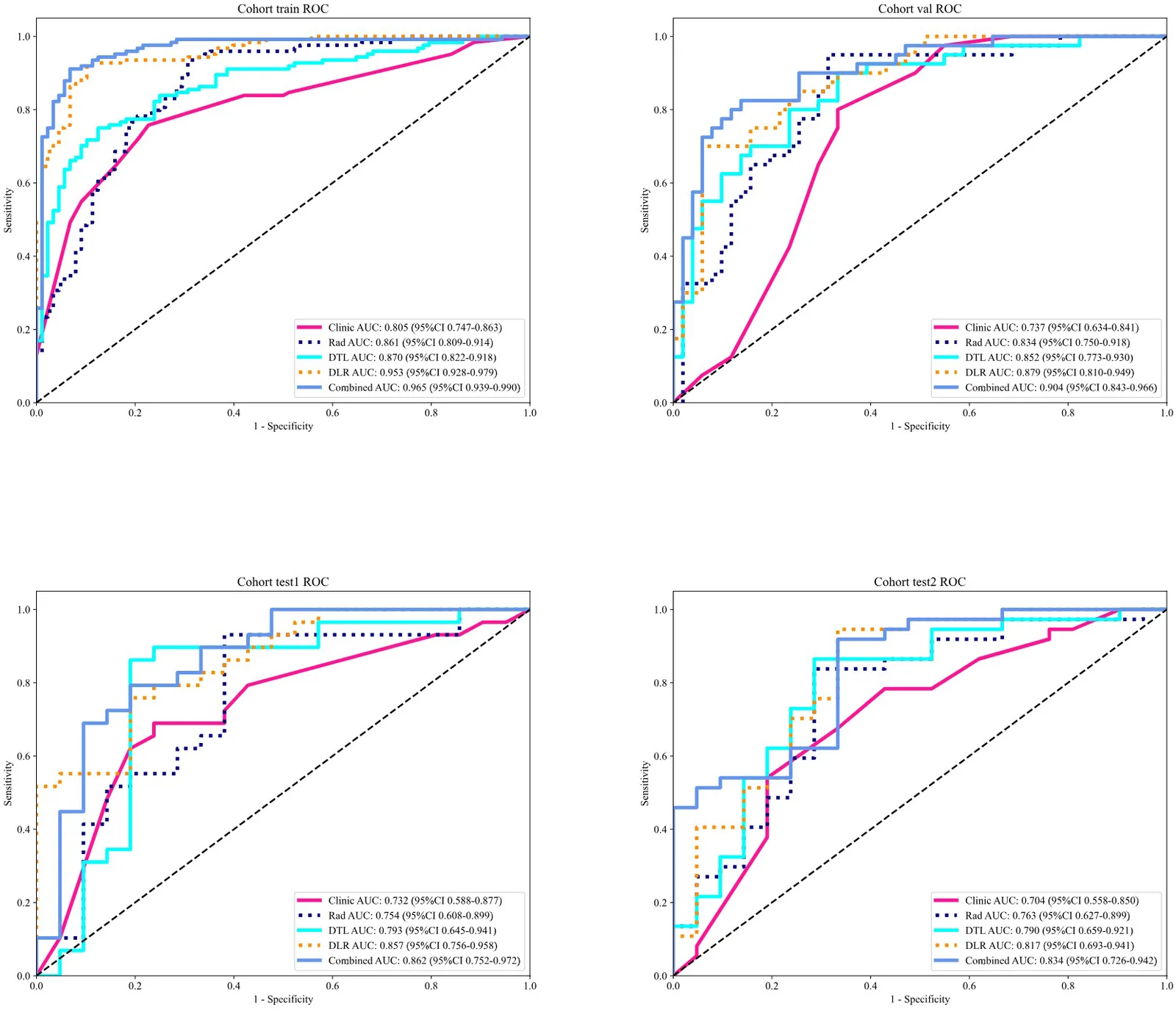
The results show the AUC values of Clinic_Sig, Rad_Sig, DTL_Sig, and DLR_Sig in the training set, internal validation set 1, external test set 1, and external test set 2. Among them, the AUC values of DLR_Sig in the training set, internal validation set, external test set 1, and external test set 2 are 0.953 (95% CI: 0.928 - 0.979), 0.879 (95% CI: 0.810 - 0.949), 0.834 (95% CI: 0.718 - 0.950), and 0.828 (95% CI: 0.707 - 0.948), respectively. All of these values are higher than those of the other groups. Therefore, the DLR model was ultimately selected, and the DLR_sig in the DLR model was combined with the 3 meaningful clinical features selected from the training set to construct a multivariate logistic regression model. The AUC value of the combined model in the training set was 0.965 (95% CI: 0.939 - 0.990), in the internal validation set was 0.904 (95% CI: 0.843 - 0.966), and in the external test set 1 and external test set 2 were 0.844 (95% CI: 0.724 - 0.964) and 0.846 (95% CI: 0.743 - 0.949), respectively. Compared with other models, the AUC value of the combined model was the highest.

**Figure 5:**
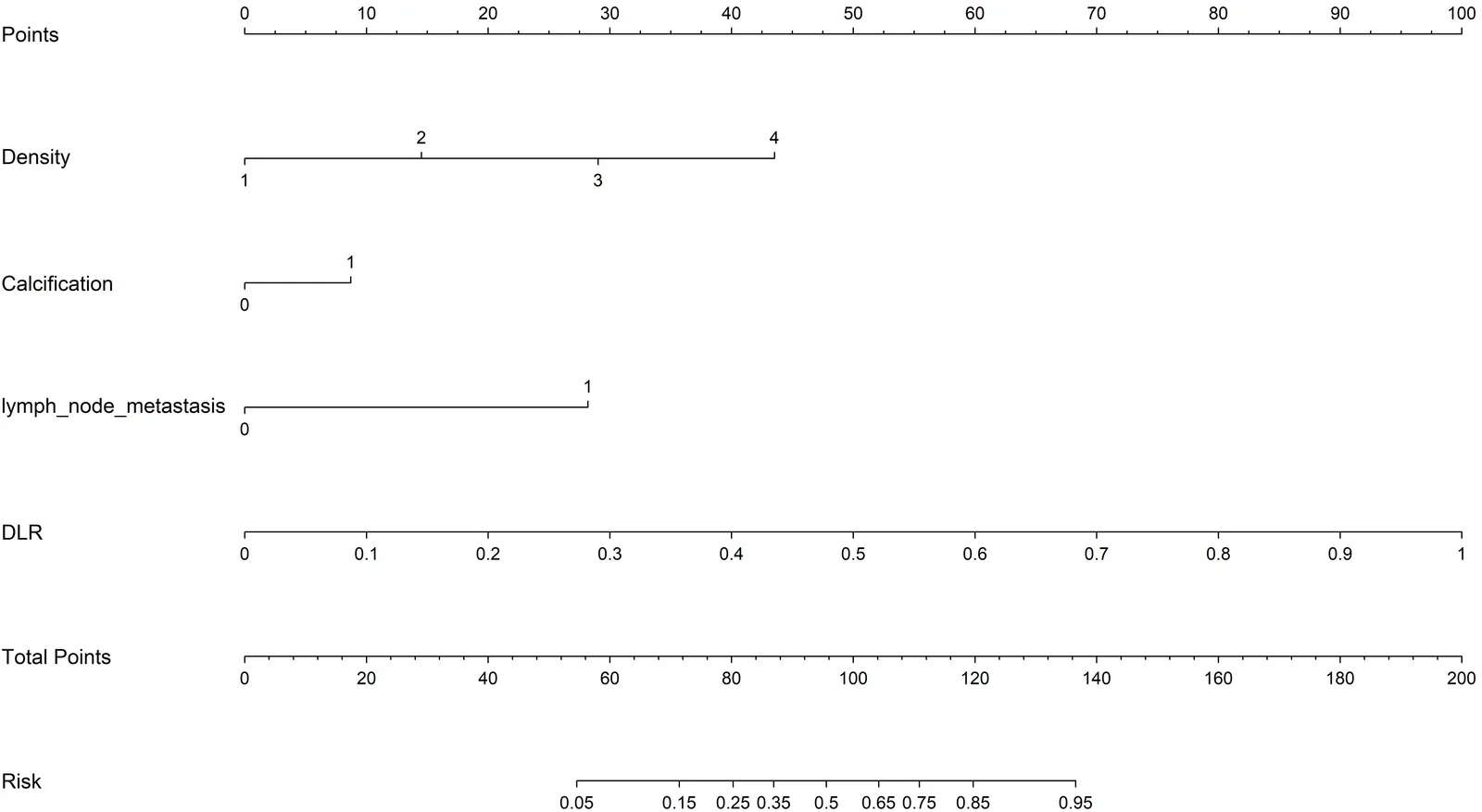
Nomogram of the clinical-DLR (radiomics-deep transfer learning) model.

**Figure 6:**
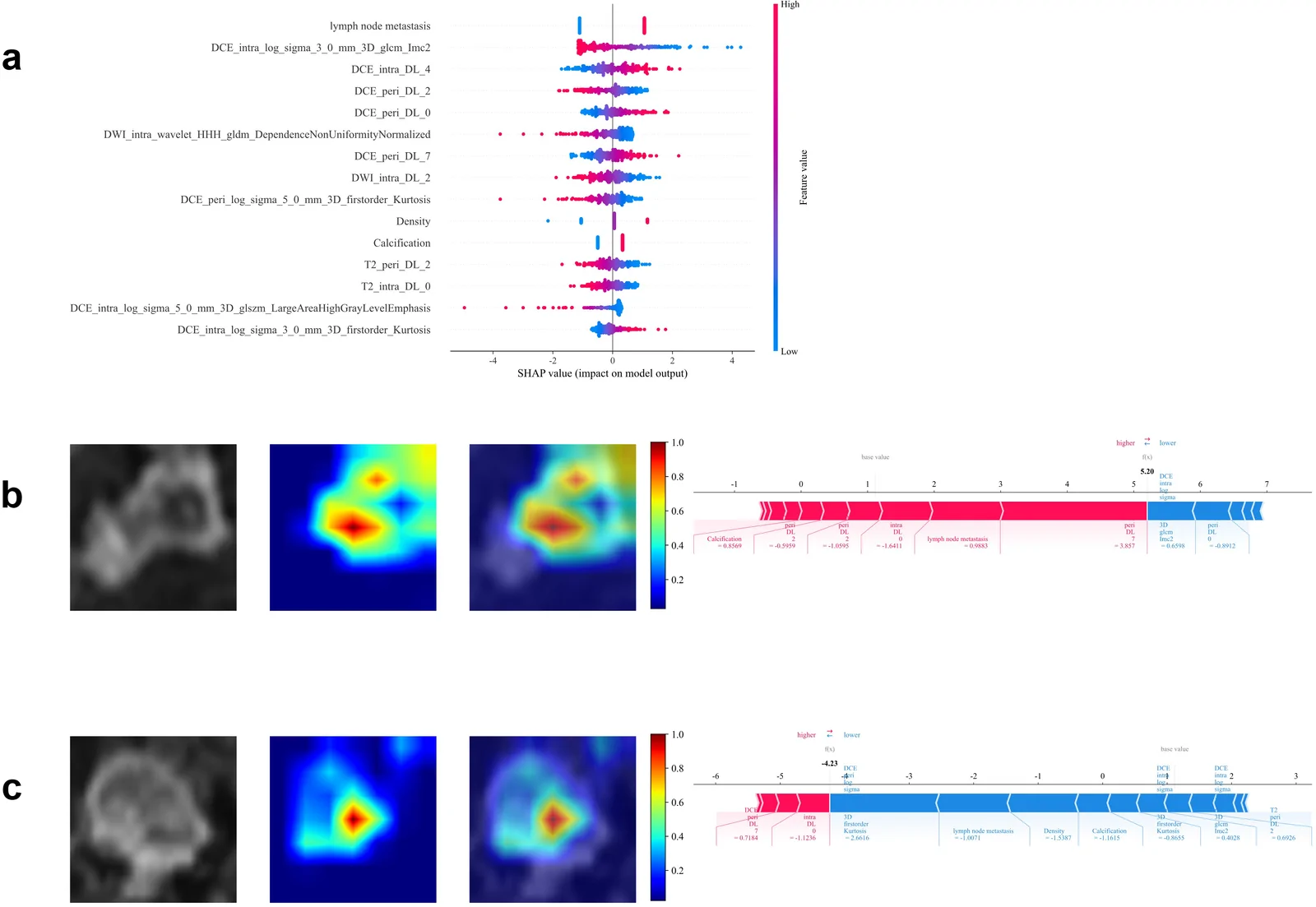
**(a)** SHAP summary plots of the clinical-radiomic-deep transfer learning model. The plot illustrates feature relevance and combined feature attributions to the model’s predictive performance. **(b, c)** Feature Heatmap and SHAP Force Diagram. **(a)** shows a HER2-positive case with a SHAP value of 5.20, exceeding the threshold (1.117), indicating a HER2-positive prediction. **(b)** shows a HER2-negative case with a SHAP value of -4.23, below the threshold (1.117). Therefore, we can assess this patient’s breast cancer as HER2-negative. The heatmap clearly shows that the orange-red regions are more extensive in positive cases than in negative cases, with the peritumoral area exhibiting a similar pattern.

The results showed that DLR_Sig obtained AUCs of 0.953, 0.879, 0.834, and 0.828 on the training set, internal validation set, external test set 1, and external test set 2, respectively, all surpassing those of other groups (**Figure 4**). A nomogram was generated by integrating the DLR_Sig metric obtained from the DLR model with three clinical significant features identified by multiple logistic regression analysis of the training set to form a combined model (**Figure 5**). In contrast to the other models, it showed the best AUC value among them. As shown in **Table 3**, Clinic_Sig’s DeLong tests against all other models showed P values < 0.05 for the training, internal validation, and external test sets, indicating predictive differences between Clinic_Sig and all other sigs in these data. Furthermore, according to DeLong’s test, even though the AUCs of the combined model, Rad_Sig, DTL_Sig, and DLR_Sig models gradually increased, they were not statistically different in terms of predictive performance across the internal validation set and external test sets 1 and 2. Calibration curves and decision curves for five signatures are presented in **Supplementary Figures 3**, **4**. The quantitative global interpretation of the ensemble model built with logistic regression was performed via SHapley additive explanations (SHAP) (**Figure 6a**). Local interpretation for individual samples was performed using Gradient-weighted class activation mapping (Grad-CAM) visualization combined with a SHAP force map that was applied for local interpretation of individual samples (**Figures 6b, c**). The performance of all models in predicting HER2 expression status is shown in **Table 4**.

**Table 3:** Delong test between models in each group.

Comparisons between groups	Training cohort	Val cohort	Test1 cohort	Test2 cohort
Clinic_Sig vs.Rad_Sig	<0.05	<0.05	<0.05	<0.05
Clinic_Sig vs.DTL_Sig	<0.05	<0.05	<0.05	<0.05
Clinic_Sig vs.DLR_Sig	<0.05	<0.05	<0.05	<0.05
Clinic_Sig vs.Combined_Sig	<0.05	<0.05	<0.05	<0.05
Rad_Sig vs.DTL_Sig	0.483	<0.05	0.341	0.685
Rad_Sig vs.DLR_Sig	<0.05	0.543	0.335	0.503
Rad_Sig vs.Combined_Sig	<0.05	0.433	0.265	0.391
DTL_Sig vs.DLR_Sig	<0.05	<0.05	0.659	0.410
DTL_Sig vs.Combined_Sig	<0.05	<0.05	0.546	0.332
DLR_Sig vs.Combined_Sig	0.132	0.594	0.470	0.366

**Table 4:** Performance comparison of all models in predicting HER-2 expression status.

Signature	Accuracy	AUC	95% CI	Sensitivity	Specificity	PPV	NPV	Cohort
Clinic	0.764	0.805	0.7468 - 0.8632	0.758	0.773	0.825	0.694	train
Rad	0.835	0.861	0.8092 - 0.9135	0.935	0.693	0.811	0.884	train
DTL	0.802	0.870	0.8218 - 0.9175	0.750	0.875	0.894	0.713	train
DLR	0.910	0.953	0.9278 - 0.9787	0.919	0.898	0.927	0.888	train
Combined	0.920	0.965	0.9393 - 0.9900	0.911	0.932	0.950	0.882	train
Clinic	0.725	0.737	0.6338 - 0.8407	0.800	0.667	0.653	0.810	val
Rad	0.802	0.834	0.7504 - 0.9182	0.950	0.686	0.704	0.946	val
DTL	0.769	0.852	0.7734 - 0.9305	0.900	0.721	0.679	0.895	val
DLR	0.835	0.879	0.8103 - 0.9485	0.700	0.941	0.903	0.800	val
Combined	0.846	0.904	0.8430 - 0.9658	0.825	0.863	0.825	0.863	val
Clinic	0.720	0.732	0.5881 - 0.8766	0.690	0.762	0.800	0.640	test1
Rad	0.860	0.791	0.6406 - 0.9423	0.724	0.782	0.806	0.667	test1
DTL	0.840	0.831	0.7011 - 0.9607	0.862	0.810	0.862	0.810	test1
DLR	0.780	0.834	0.7182 - 0.9501	0.759	0.805	0.846	0.708	test1
Combined	0.800	0.844	0.7236 - 0.9644	0.793	0.828	0.852	0.739	test1
Clinic	0.707	0.704	0.5580 - 0.8500	0.784	0.571	0.763	0.600	test2
Rad	0.741	0.804	0.6793 - 0.9295	0.828	0.703	0.842	0.750	test2
DTL	0.810	0.803	0.6737 - 0.9325	0.865	0.762	0.865	0.762	test2
DLR	0.828	0.828	0.7070 - 0.9481	0.892	0.714	0.846	0.789	test2
Combined	0.845	0.846	0.7426 - 0.9486	0.946	0.811	0.833	0.875	test2

PPV, positive prediction value; NPV, negative prediction value; AUC, area under the curve.

## 4 Discussion

This study created a multiparametric MRI-based radiomics-deep learning fusion model. By combining the multi-dimensional features of intratumoral and peritumoral areas, along with clinical features, it could accurately predict HER2 status in breast cancer. The model had an AUC of 0.965 (95% CI: 0.939–0.990) for the training set and 0.904 (95% CI: 0.843–0.966) for the internal validation set. The AUCs on external test sets 1 and 2 were 0.844 (95% CI: 0.724–0.964) and 0.846 (95% CI: 0.743–0.949), respectively. These results were comparable to those of the multimodal model of Huang et al. (20) (AUC = 0.840) and were substantially better than those of traditional monomodality models. This result demonstrates that using a combination of multiple modalities, such as imaging and data from deep neural networks, has advantages over existing techniques for classifying different types of breast cancers, such as HER2 subtype, without direct tumor tissue sampling, which may require surgery. Instead, these approaches can use less invasive, non-surgical methods.

### 4.1 Clinical imaging feature predictive value of HER2 expression level in breast cancer

Recent studies show that DCE-MRI, T2WI, and DWI sequences provide hemodynamic (21), anatomical (22), and cell density (23) information, respectively. This multi-parameter fusion strategy captures the full spectrum of tumor heterogeneity across the “function-structure-cell” axis. Moreover, it should be mentioned that choosing early enhancement features from DCE-MRI could represent HER2-induced angiogenesis activity and thus significantly improve the model performance (24–27). Growing evidence indicates that peritumoral tissue is an essential component in tumor evolution; alterations such as edema, infiltration of inflammatory cells, and neovascularization are closely linked to the aggressiveness of HER2-positive tumors (28, 29). This study found that deep learning features from the peritumoral region made a significant contribution to model prediction. Of the 12 finally selected radiomics and deep learning features, 5 were peritumoral features, including four deep learning features. Grad-CAM heatmaps also visually showed the connection between the tumor’s peritumoral area and its HER2 level, which was consistent with the findings discussed above. This suggests that deep learning has the ability to identify microenvironmental changes that cannot be seen by the human eye, providing a new way of understanding how HER2+ breast cancer behaves. In recent years, much work has been done to predict HER2 status in breast cancers based on full-field digital mammography (FFDM) images. Studies have shown that tumor tissue can change the microenvironment by secreting bone morphogenetic protein 2 (BMP-2), thereby causing microcalcification. Breast mammography is highly sensitive for detecting microcalcifications in breast cancer, and there is a clear association between HER2-positive or HER2-overexpressing breast cancer and microcalcifications (30, 31). This study also found a very strong link between microcalcifications seen in FFDM images and HER2 overexpression, consistent with previous studies. Thus, FFDM serves as an important supplementary tool for predicting HER2 status. Microcalcifications not only indicate tumor malignancy but are also closely related to abnormal calcium-phosphorus metabolism caused by the activation of the HER2 signaling pathway. This finding offers a novel perspective for understanding the biological traits of HER2-positive breast cancer.

### 4.2 Predictive value of radiomics and deep learning models for HER2 expression levels in breast cancer

Radiomics can quantitatively characterize tumor heterogeneity by obtaining high-throughput quantitative image features. This study obtained 7,182 radiomics features from DCE-MRI, T2WI, and DWI. After rigorous screening, we retained the top 18 most predictive features to form a radiomics signature. The model showed good performance in both the training set and the validation set (AUC: 0.79–0.86), which was similar to that reported in some recent studies. For example, Yue et al. (32) obtained an AUC of 0.867 when they used a DCE-MRI-based radiomics model to differentiate HER2 status; Fang et al. (33) reported that a model combining radiomics and clinical features had an AUC of 0.84. This study also found that texture and wavelet features had the greatest contribution to predicting HER2 status, which might reflect the greater structural complexity caused by HER2 overexpression in the tumor microenvironment. The advantage of radiomics features is that they are highly interpretable, allowing and intuitive association between imaging characteristics and tumor biological behavior.

Radiomics uses manually designed features, whereas deep learning learns complicated patterns in images by using convolutional neural networks. This study used a pretrained ResNet50 model to extract 2,048 deep learning features from the intratumoral and peritumoral regions. The results showed that the deep learning model maintained good generalization ability in the external test set (AUC: 0.80–0.83). This finding is consistent with that of Guo et al. (34), who reported an AUC of 0.87 when predicting HER2 status using a DCE-MRI-based deep learning model. The particular strength of deep learning is its capacity to identify subtle image patterns that cannot be easily recognized by the human eye, such as very slight infiltration of the tumor edge and delicate changes in structures around the tumor. These could relate to the local microenvironment alterations caused by HER2 signaling pathway activation. Notably, this study found that the deep learning model showed particular strengths in extracting features from peritumoral areas. Grad-CAM visualization revealed that the activation of HER2-positive tumor areas in adjacent regions is unique, which supports earlier studies showing the involvement of the tumor microenvironment in HER2 signaling. Studies show that HER2 overexpression affects peritumoral matrix remodeling by modulating tumor-associated fibroblast activity, and deep learning can uniquely capture these microstructural changes (35).

The study created a DLR model based on 18 selected radiomics features and 38 deep learning features, which showed substantially better performance across all evaluation metrics than the single-model approaches. The DLR model AUC was 0.953 in the training set, 0.879 in the validation, and 0.828–0.834 in the external test sets. These results indicate that the model has good generalizability. This improvement arises from both components: radiomics provides interpretable information about certain imaging characteristics related to HER2 status (such as heterogeneity of tumor textures and enhancement patterns), whereas deep learning identifies complex relationships in high-dimensional spaces and minor image modifications that traditional features cannot capture. When compared with other studies, the integration strategy employed in this research demonstrates distinct advantages. Fan et al. (36) obtained an AUC of 0.848 for predicting HER2 status via a multimodal image fusion model. In the present study, we improved the performance of our model even further by selecting and combining the most informative features. Moreover, Quan et al.’s ultrasound-based deep learning models (37) and Yang et al.’s CT-based radiomics models (38), although also achieving good results, do not provide as much feature information or biological interpretability as our multiparametric MRI fusion model.

### 4.3 Limitations and perspectives

This study has several limitations. First, the retrospective design might have introduced selection bias and should be addressed through future large-sample prospective multicenter studies. Patients undergoing neoadjuvant therapy were not included; whether the model is useful for evaluating treatment response requires further exploration. For FFDM analysis, the current analysis focuses on microcalcification characteristics. Future work could look at how other images, such as breast density or tumor shape, affect the prediction of HER2 status. Lastly, the current region of interest segmentation also contains manual steps; we will explore more advanced automated segmentation algorithms to improve efficiency and consistency.

## 5 Conclusion

Overall, this study created a high-performance and broadly applicable MRI-FFDM integrated model with multiple parameters through the complementary integration of multiple parameter sequences, comprehensive analysis, exploration of peritumoral microenvironmental traits, assessment of FFDM microcalcification features, and integration of the advantages of radiomics and deep learning. The model can serve as an effective way to non-invasively and precisely predict HER2 status in breast cancer patients.

## Data Availability

The original contributions presented in the study are included in the article/**Supplementary Material**. Further inquiries can be directed to the corresponding authors.
