# Supplementary material for: Integrating intratumoral and peritumoral radiomics with deep transfer learning from multiparametric MRI for preoperative prediction of HER2 status in breast cancer: a multicenter study
Source: Front Oncol. 2026 Aug 28;16:1790816. doi: 10.3389/fonc.2026.1790816 (PMC13573207; doi:10.3389/fonc.2026.1790816)
Supplement: Supplementary file 1 [file DataSheet1.docx]

**Supplementary notes：Deep transfer learning detailed process**

Transfer Learning: In this study, we explored the performance of prominent networks such as ResNet50, ResNet101, and DenseNet121 to enhance the performance of traditional CNN-based models. Additionally, we conducted comparative analyses of these models to identify the most suitable algorithm for our specific research requirements.

Hyperparameters: In our study, to ensure the model's effectiveness across various patient populations with notable variability, we implemented transfer learning. This process involved initializing the model with pre-trained weights from the ImageNet database, enhancing its adaptability to diverse datasets. A critical aspect of our approach was the meticulous adjustment of the learning rate to foster better generalization across datasets. For this purpose, we employed the cosine decay learning rate strategy, defined as follows:

$\text{η}_{\text{t}}\text{=}\text{η}_{\text{min}}^{\text{i}}\text{+}\frac{\text{1}}{\text{2}}\text{(}\text{η}_{\text{max}}^{\text{i}}\text{−}\text{η}_{\text{min}}^{\text{i}}\text{)(1+}\text{cos}\text{(}\frac{\text{T}_{\text{cur}}}{\text{T}_{\text{i}}}\text{π}\text{))}$，Specifically, $\text{η}_{\text{min}}^{\text{i}}$=0，$\text{η}_{\text{max}}^{\text{i}}$=0.01,and $\text{T}_{\text{i}}$=50 denote the minimum learning rate, maximum learning rate, and number of iterations in the training process, respectively. DenseNet121 selects the nth layer from the features.norn5 layers, while other models choose the average pooling layer to extract deep features.Other essential hyperparameters include the use of Stochastic Gradient Descent (SGD) as the optimizer and softmax cross-entropy for the loss function.

**Supplementary table 1: Prediction performance of three pre-trained Convolutional Neural Network (CNN) models for the DTL_Sig**

| Model | AUC | 95% CI | Sensitivity | Specificity | MRI Modal | Cohort |
| --- | --- | --- | --- | --- | --- | --- |
| *resnet50* | *0.813* | *0.7544-0.8717* | *0.830* | *0.705* | *DCE* | *Training cohort* |
| *resnet50* | *0.739* | *0.6688-0.8088* | *0.689* | *0.720* | *DCE* | *Test cohort* |
| *resnet50* | *0.766* | *0.7031-0.8295* | *0.815* | *0.591* | *T2WI* | *Training cohort* |
| *resnet50* | *0.708* | *0.6347-0.7812* | *0.811* | *0.581* | *T2WI* | *Test cohort* |
| *resnet50* | *0.780* | *0.7169-0.8439* | *0.750* | *0.727* | *DWI* | *Training cohort* |
| *resnet50* | *0.711* | *0.6376-0.7835* | *0.726* | *0.634* | *DWI* | *Test cohort* |
| *resnet101* | *0.742* | *0.6743-0.8088* | *0.661* | *0.736* | *DCE* | *Training cohort* |
| *resnet101* | *0.679* | *0.6036-0.7536* | *0.524* | *0.800* | *DCE* | *Test cohort* |
| *resnet101* | *0.631* | *0.5552-0.7067* | *0.645* | *0.625* | *T2WI* | *Training cohort* |
| *resnet101* | *0.581* | *0.5054-0.6650* | *0.774* | *0.409* | *T2WI* | *Test cohort* |
| *resnet101* | *0.645* | *0.5694-0.7199* | *0.567* | *0.671* | *DWI* | *Training cohort* |
| *resnet101* | *0.582* | *0.4991-0.6643* | *0.754* | *0.407* | *DWI* | *Test cohort* |
| *densenet121* | *0.696* | *0.6235-0.7675* | *0.701* | *0.635* | *DCE* | *Training cohort* |
| *densenet121* | *0.559* | *0.4766-0.6418* | *0.525* | *0.617* | *DCE* | *Test cohort* |
| *densenet121* | *0.641* | *0.5668-0.7159* | *0.551* | *0.694* | *T2WI* | *Training cohort* |
| *densenet121* | *0.574* | *0.4908-0.6563* | *0.720* | *0.469* | *T2WI* | *Test cohort* |
| *densenet121* | *0.734* | *0.6647-0.8040* | *0.622* | *0.765* | *DWI* | *Training cohort* |
| *densenet121* | *0.634* | *0.5441-0.7239* | *0.731* | *0.545* | *DWI* | *Test cohort* |

**Supplementary Fig.1 The Ratio of each group of handcrafted features.**

**Supplementary Fig.2 Feature selection using the LASSO binary logistic regression model:**

**(a) all features' corresponding p-value results.**

**(b) LASSO coefficient profiles of the features show that are drawn at the value selected using 10 fold cross validation,and the optimal λ results in 49 nonzero coefficients.**

**(c) MSE(mean standard error) of 10 fold cross validation.**

**Supplementary Fig.3 Calibration Curves of Different Models**（The calibration plot of the joint model most closely approximates the ideal curve, indicating excellent agreement between the actual probability of predicting HER-2 expression status and the predicted probability.）

**Supplementary Fig.4 Decision Curve Analysis of Different Models**（In terms of overall net benefit provided by the five models, the radiomics model, deep learning model, radiomics-deep learning model, and combined model all outperformed the clinical model, with the combined model demonstrating the highest decision-making efficacy.）
